# Self-Complementary Adeno-Associated Virus Vectors Improve Transduction Efficiency of Corneal Endothelial Cells

**DOI:** 10.1371/journal.pone.0152589

**Published:** 2016-03-29

**Authors:** Anja K. Gruenert, Marta Czugala, Chris Mueller, Marco Schmeer, Martin Schleef, Friedrich E. Kruse, Thomas A. Fuchsluger

**Affiliations:** 1 Department of Ophthalmology, University of Erlangen-Nurnberg, Erlangen, Germany; 2 Department of Pediatrics and Horae Gene Therapy Center, University of Massachusetts Medical School, Worcester, Massachusetts, United States of America; 3 PlasmidFactory GmbH & Co. KG, Bielefeld, Germany; National Institutes of Health, UNITED STATES

## Abstract

Transplantation of a donor cornea to restore vision is the most frequently performed transplantation in the world. Corneal endothelial cells (CEC) are crucial for the outcome of a graft as they maintain corneal transparency and avoid graft failure due to corneal opaqueness. Given the characteristic of being a monolayer and in direct contact with culture medium during cultivation in eye banks, CEC are specifically suitable for gene therapeutic approaches prior to transplantation. Recombinant adeno-associated virus 2 (rAAV2) vectors represent a promising tool for gene therapy of CEC. However, high vector titers are needed to achieve sufficient gene expression. One of the rate-limiting steps for transgene expression is the conversion of single-stranded (ss-) DNA vector genome into double-stranded (ds-) DNA. This step can be bypassed by using self-complementary (sc-) AAV2 vectors. Aim of this study was to compare for the first time transduction efficiencies of ss- and scAAV2 vectors in CEC. For this purpose AAV2 vectors containing enhanced green fluorescent protein (GFP) as transgene were used. Both in CEC and in donor corneas, transduction with scAAV2 resulted in significantly higher transgene expression compared to ssAAV2. The difference in transduction efficiency decreased with increasing vector titer. In most cases, only half the vector titer of scAAV2 was required for equal or higher gene expression rates than those of ssAAV2. In human donor corneas, GFP expression was 64.7±11.3% (scAAV) and 38.0±8.6% (ssAAV) (p<0.001), respectively. Furthermore, transduced cells maintained their viability and showed regular morphology. Working together with regulatory authorities, a translation of AAV2 vector-mediated gene therapy to achieve a temporary protection of corneal allografts during cultivation and transplantation could therefore become more realistic.

## Introduction

Corneal endothelial cells (CEC) form a monolayer on the posterior surface of the cornea and are of fundamental relevance for vision. Their pump function is essential to maintain corneal transparency by deswelling the corneal stroma [1–3]. As human CEC practically do not proliferate [4], their decrease below a critical minimum unavoidably results in swelling of the cornea leading to corneal opaqueness and ultimately to functional blindness. Moreover, both viability and density of endothelial cells are the most relevant parameters in evaluation of donor corneas processed in eye banks to supply the 300,000+ transplantations performed worldwide each year. These criteria define the suitability of donor corneas for transplantation and further determine the probability of graft survival and risk of re-transplantation [5, 6].

Thanks to distinct properties the corneal endothelium is a very attractive target for gene and cell therapeutic approaches: 1. its monolayer structure results in direct contact of every single cell to the culture medium *ex vivo* or to the aqueous humour of the anterior ocular chamber *in vivo*; 2. its avascularity and immune privilege reduce the risk of immune reactions [7, 8]; 3. compulsory processing of donor corneas in eye banks prior to engraftment over one to four weeks opens the possibility of *ex vivo* gene therapy. Possible applications of gene delivery to the corneal endothelium include immune modulation to prevent corneal graft rejection and improving corneal allograft survival by protecting the endothelial monolayer from culture- and transplant-associated apoptosis [9, 10].

Recombinant adeno-associated virus (rAAV) vectors provide a promising alternative to other viral vectors and gene delivery systems, as they are nonpathogenic, replication-deficient and transduce both dividing and non-dividing cells [11, 12]. Additionally, in contrast to lentiviral vectors which we have used in our previous studies [13, 14], AAV is non-integrating, thus increasing its safety and conforming to regulatory requirements. Various cell types and tissues can be efficiently transduced by AAV including liver [15], muscle [16], lung [17], CNS [18] and bone marrow [19]. In the eye, practically all studies on AAV-mediated gene therapy were performed to establish therapies targeting gene defects in the retina [20–23]. Successful transduction, however, has also been demonstrated in the trabecular meshwork in glaucoma research [24–26], in corneal stroma [27, 28] and in corneal endothelium [29–31].

In conventional AAV vectors, the genome is packaged as a linear single-stranded (ss) DNA molecule with a length of approximately 4.7 kb. These single strands occur either in plus- or in minus-form. Prior to gene expression, it is necessary to convert ssDNA into double-stranded (ds) DNA either by strand annealing of one plus- and one minus-strand or by *de novo* synthesis of DNA. This is known to be one of the rate-limiting steps for efficient transduction [32–34]. Application of self-complementary (sc) AAV vectors allows a complete bypass of this issue as these vectors contain a dimeric inverted repeat genome able to fold into dsDNA [32]. However, packing all complementary bases in the same virus particle limits the coding capacity of scAAV to about half of that of ssAAV [32].

Previous studies have shown increased transduction efficiencies with scAAV [35–39]. As the relative advantage of scAAV in comparison to ssAAV varies among different cell types, the aim of this study was to analyze whether and by which extent transduction efficiencies in CEC could be increased by the use of scAAV. This is of high relevance for a potential use of AAV in *ex vivo* corneal gene therapy during cultivation of corneas in eye banks. Furthermore, CEC viability following GFP insertion by the different AAV vector types was controlled.

## Methods

### Cell culture / organ culture conditions

The human corneal endothelial cell line (HCEC-12, DMSZ No. ACC-646) was acquired from Leibniz Institute DSMZ-German Collection of Microorganisms and Cell Cultures (Braunschweig, Germany) in 2012. Passages 18 to 22 were used for all experiments. Cells were trypsinized (Trypsin/EDTA Solution 0.25% / 0.02% in PBS; Biochrom, Berlin, Germany) and seeded in a 48-well plate containing 500 μl of culture medium at 60% confluency. Culture medium was composed of Minimal Essential Medium (MEM; Sigma-Aldrich, St. Louis, MO, USA) with 5% fetal bovine serum (FBS) and 0.5% gentamicin (Sigma-Aldrich). Cells were incubated in a humidified atmosphere at 37°C and 5% CO_2._ To maintain the cells, culture medium was changed every other day.

Research-grade human donor corneas with intact endothelium cultivated at 4°C in Optisol GS (Bausch&Lomb, Bridgewater, NJ, USA) were obtained from Indiana Lions Eye Bank (Indianapolis, IN, USA; n = 4). Prior to transduction, corneoscleral buttons were transferred to 37°C organ-culture and pre-incubated in 4 ml culture medium containing FBS, epidermal growth factor (EGF) and bovine pituitary extract (BPE), similar to Chen’s medium [40].

### Transduction of CEC

Ss- and scAAV2 vectors containing the GFP gene driven by a cytomegalovirus (CMV) promoter were kindly provided by Prof. Dr. H. Büning (Center for Molecular Medicine Cologne (CMMC), Laboratory for AAV vector development, University of Cologne, Cologne, Germany). Vector preparations were produced by plasmid co-transfection of Human Embryonic Kidney (HEK) 293 cells as previously described [41]. As plasmids, either pssGFP or pscGFP were used together with pXX [42] and pRC [43]. Cells were harvested, centrifuged, lysed and treated with benzonase. The lysate was purified using an iodixanol density gradient. Vector suspensions were obtained by extracting the 40% iodixanol phase and aliquoted without further dilution or concentration.

24 hours after seeding of HCEC-12 cells, culture medium was replaced by 300 μl of fresh medium and vector suspension was added according to the desired vector titer (day 0).

Human donor corneas with intact endothelium and high cell count unsuitable for transplantation in humans were used according to the Erlangen University Ethic Committee approval and to the Declaration of Helsinki. Each cornea was cut into six pieces and put into separate wells of a 48-well plate. 500 μl of fresh medium and the respective amount of vector suspension were added (day 0).

HCEC-12 cells and human corneal tissue were incubated with the vector suspension for 48 hours at 37°C and 5% CO_2_. After washing off the vector (day 2), culture media were replaced every other day.

### Evaluation of transgene expression

GFP expression in HCEC-12 cells was analyzed by flow cytometry (FACS Canto II with FACSDiva software, BD Biosciences, San Jose, CA, USA) directly after the transduction period (day 2) as well as on days 5, 8, 12, 16, 22 and 28. Three independent experiments were performed for each vector titer and for each point of time. Supernatants and trypsinized cells were transferred to 15ml tubes on ice followed by centrifugation (3 minutes, 1200 rpm) and resuspension of the cells in FACS buffer (PBS with 10% FCS). A total of 20,000 cells per sample were analyzed.

GFP expression in corneal tissue (n = 4) was evaluated by confocal microscopy at 200X magnification on day 6 (Zeiss LSM 780 Confocal Microscope, Jena, Germany; six random microscopic fields per corneal piece). After washing (PBS containing Ca^2+^ and Mg^2+^) and fixation (4% paraformaldehyde in PBS, 15 minutes), cell membranes were marked using rabbit anti-ZO-1 antibodies diluted 1:200 (Thermo Fisher Scientific, Waltham, MA, USA) and goat-anti-rabbit IgG (H+L) conjugated to Alexa Fluor 647 (Jackson ImmunoResearch Laboratories, West Grove, PA, USA) at a dilution of 1:500. Subsequently, corneal samples were stained with DAPI (1 mg/ml, dilution 1:1500 in PBS) and mounted on glass slides endothelial side up with Vectashield mounting medium (Vector Laboratories, Burlingame, CA, USA).

### Cell viability and metabolism evaluation

To assess viability of transduced CEC, HCEC-12 cells were harvested at the end of transduction (day 2).Three independent experiments were performed for each vector titer (25,000, 50,000 and 100,000 genomic particles of infection (GOI = AAV-capsids containing vector DNA). Supernatants and trypsinized cells were transferred to 15ml tubes on ice followed by centrifugation (3 minutes, 1200 rpm). After centrifugation, cells were resuspended in 200 μl FACS-Buffer containing 2.5 μg/ml PO-PRO^TM^-1 iodide (Thermo Fisher Scientific) and 5μg/ml 7-AAD (7-Aminoactinomycin D, Sigma-Aldrich). PO-PRO^TM^-1 and 7-AAD are DNA-intercalating dyes which are impermeant to membranes of living cells. While PO-PRO^TM^-1 can permeate through slightly impaired cell membranes of apoptotic cells 7-AAD is only permeant to dead cells. Therefore, these dyes are used to discriminate vital cells with intact cell membrane from apoptotic and dead cells allowing quantification by flow cytometry. Non-transduced HCEC-12 cells seeded on the same day served as negative controls. Flow cytometry was performed using FACS Canto II with FACS DIVA software. A total of 20,000 cells per sample were analyzed.

To study the metabolic state of transduced CEC, MTT (3-(4,5-dimethylthiazol-2-yl)-2,5-diphenyltetrazolium bromide)-assay was performed immediately after transduction (day 2). Culture medium was removed and replaced by fresh culture medium containing 0.5 mg/ml MTT (Sigma Aldrich). Viable, metabolically active cells reduce MTT into purple colored formazan, which can be measured quantitatively in terms of absorbance. After incubation at 37°C and 5% CO_2_ for 2 hours, MTT solution was removed and resulting formazan was dissolved in 300 μl DMSO (Sigma Aldrich). Colorimetric analysis was performed using a spectrophotometer (Multiskan Spectrum, Thermo Fisher Scientific).

In human donor corneas DNA strand breaks of apoptotic cells were labeled using TUNEL (terminal deoxynucleotidyl transferase (TdT)-mediated deoxyuridine triphosphate (dUTP)-X nick end labeling)-assay (In Situ Cell Death Detection Kit, TMR red, Roche Diagnostics GmbH, Mannheim, Germany) according to the manufacturer’s instructions. Evaluation was performed using confocal microscopy.

### Statistical analysis

Statistical analysis was performed using GraphPad Prism (Version 6.01). Means and standard deviations (SD) were calculated. Comparisons between two groups were performed using Student’s t-test or Wilcoxon test. Multiple comparisons were performed using one-way ANOVA test. P-values lower than 0.05 were considered statistically significant (* = p<0.05; ** = p<0.01; *** = p<0.001).

## Results

### GFP expression in HCEC-12 cells

To study transduction efficiencies following transduction with ss- and scAAV2, HCEC-12 cells were treated with vector titers ranging from 1,000 to 100,000 GOI. In general, the amount of GFP expressing cells detected by flow cytometry increased with increasing vector titer. Use of scAAV2 resulted in significantly higher transgene expression levels compared to ssAAV2.

**Fig 1:**
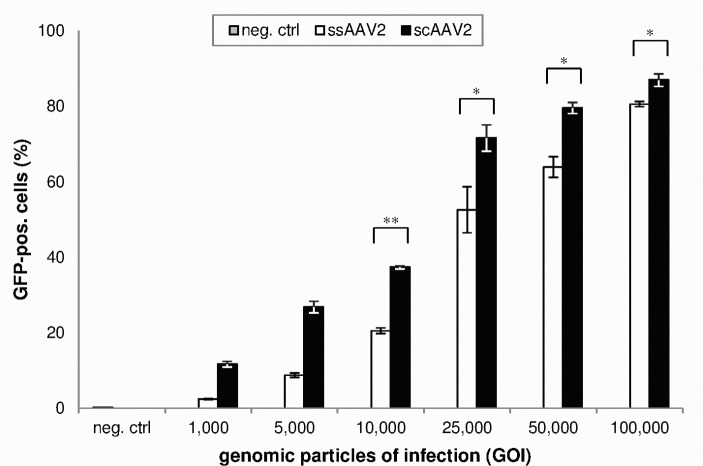
GFP expression of HCEC-12 cells five days after transduction (flow cytometry). Means and standard deviations of three independent experiments are shown. GFP expression after transduction with scAAV was significantly higher compared to ssAAV2 (* = p<0.05, ** = p<0.01).

Fig 1 shows the amount of GFP expressing HCEC-12 cells 5 days after transduction with equal titers of ss- or scAAV2. The difference between transduction efficiencies of ss- and scAAV2 was greater for lower vector titers and smaller for higher vector titers. Using 1,000 GOI, the amount of GFP expressing cells on day 5 was 4.8-fold higher with scAAV2 compared to ssAAV2 (p = 0.25). Using 10,000 GOI, scAAV2 lead to a 1.8-fold higher transduction rate (p = 0.002), and using 100,000 GOI, the transduction rate with scAAV2 was merely 8% higher than with ssAAV2 (p = 0.04).

**Fig 2:**
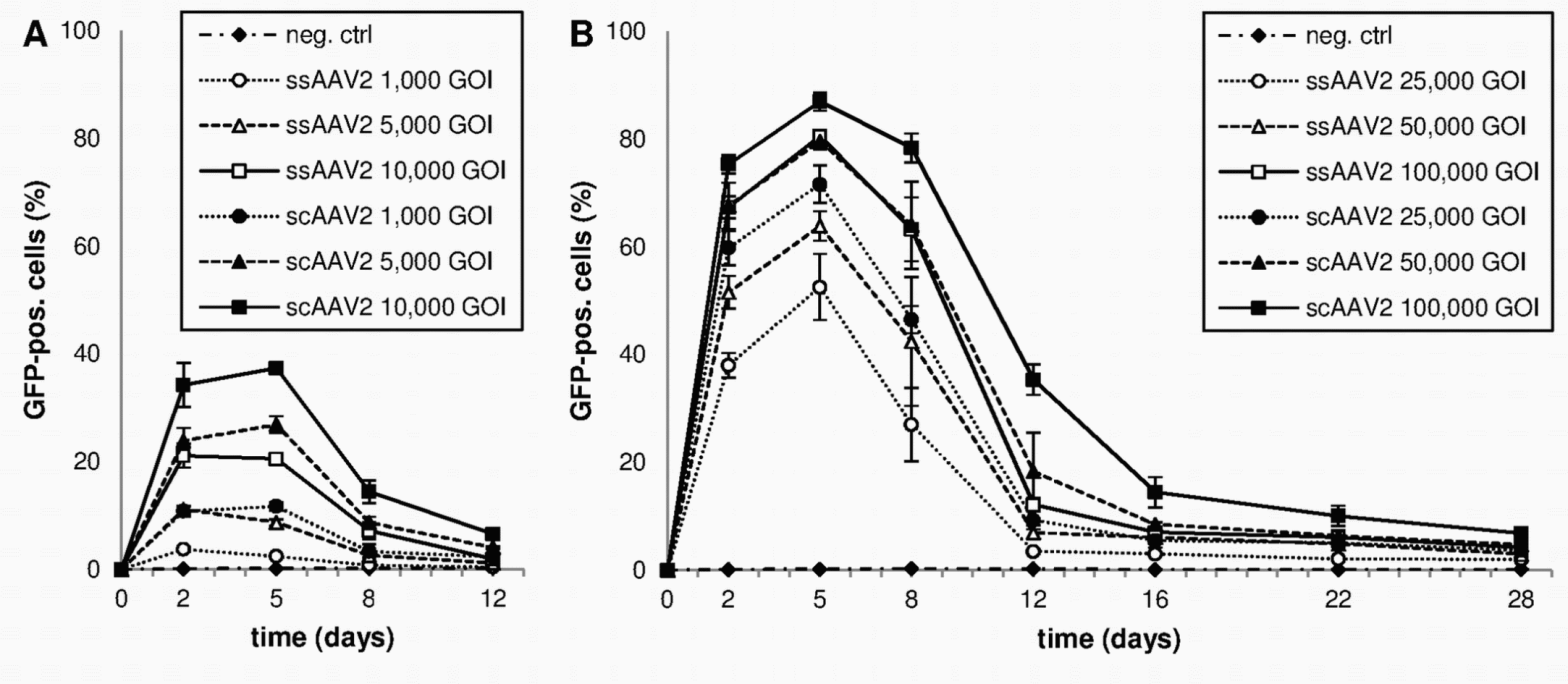
GFP-expression levels of transduced HCEC-12 cells over time (flow cytometry). Means and standard deviations of three independent experiments are shown. (A) vector titers 1,000-10,000 GOI; (B) vector titers 25,000–100,000 GOI.

To determine the kinetics of GFP expression, flow cytometry analyses were performed over a period of 28 days. Results are presented in Fig 2(A) (vector titers 1,000–10,000 GOI) and Fig 2(B) (vector titers 25,000–100,000 GOI). Both vectors yielded a rapid onset of transgene expression. 48 hours after transduction, GFP expression levels in cells transduced with ssAAV2 ranged from 3.7% ± 0.4% (1,000 GOI) to 67.3% ± 2% (100,000 GOI), while GFP expression levels following transduction with scAAV2 ranged from 10.8% ±1% (1,000 GOI) to 75.3% ± 1,7% (100,000 GOI). For most titers, the GFP expression peak was reached on day 5. The highest transgene expression levels observed were 80.5% (ssAAV2) and 86.9% (scAAV2), respectively (Fig 2(B)).

After transduction with 1,000 to 10,000 GOI ssAAV2, GFP expression levels peaked on day 2 and decreased afterwards (Fig 2(A)). In scAAV2, however, transduction with equal vector titers lead to increasing expression levels until day 5. Between days 5 and 8 the amount of GFP expressing cells decreased again by factor 3 (2.6–3.4). After 12 days, expression levels were below 5%.

Transductions with 25,000 to 100,000 GOI resulted in a more moderate loss of GFP expression between days 5 and 8 (Fig 2(B)). Use of higher vector titers resulted in sustained GFP expression compared to lower vector titers: While cells transduced with 100,000 GOI lost 10% (scAAV2) to 22% (ssAAV2) of GFP expression, cells transduced with 25,000 GOI of ssAAV2 showed almost 50% reduction in GFP-positivity until day 8.

Furthermore, GFP expression was prolonged by using scAAV. 12 days after transduction with 100,000 GOI scAAV2, 35.3% of the cells still expressed the transgene. At that time, maximum expression level for ssAAV2 was 12.1%. Afterwards, expression levels in both groups further decreased and dropped below 7% by day 28.

**Fig 3:**
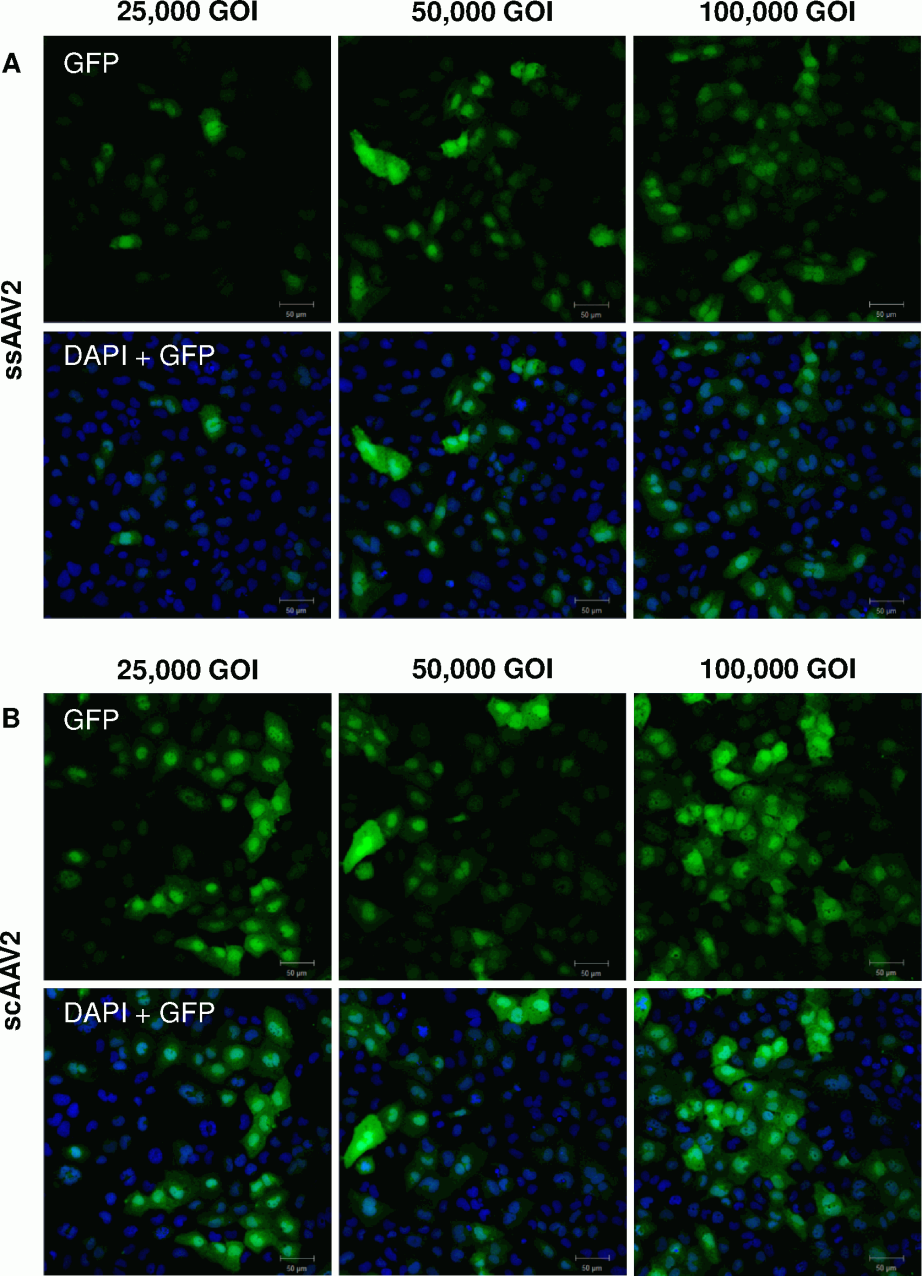
Confocal microscopy images of HCEC-12 cells transduced with different vector titers two days after transduction. (A) cells transduced with ssAAV2; (B) cells transduced with scAAV2.

In almost all cases, application of only half the titer of scAAV resulted in equal or higher transgene expression levels compared to ssAAV2 (Fig 2(A) and 2(B)). For lower vector titers, the difference of dose-response relationship and therefore transduction efficiency was even more evident: 1,000 GOI of scAAV2 yielded higher expression levels than 5,000 GOI of ssAAV2 (p = 0.25). To confirm and visualize these findings, confocal microscopy of HCEC-12 cells seeded on chamber slides was performed (Fig 3).

### GFP expression in CEC of human corneas

**Fig 4:**
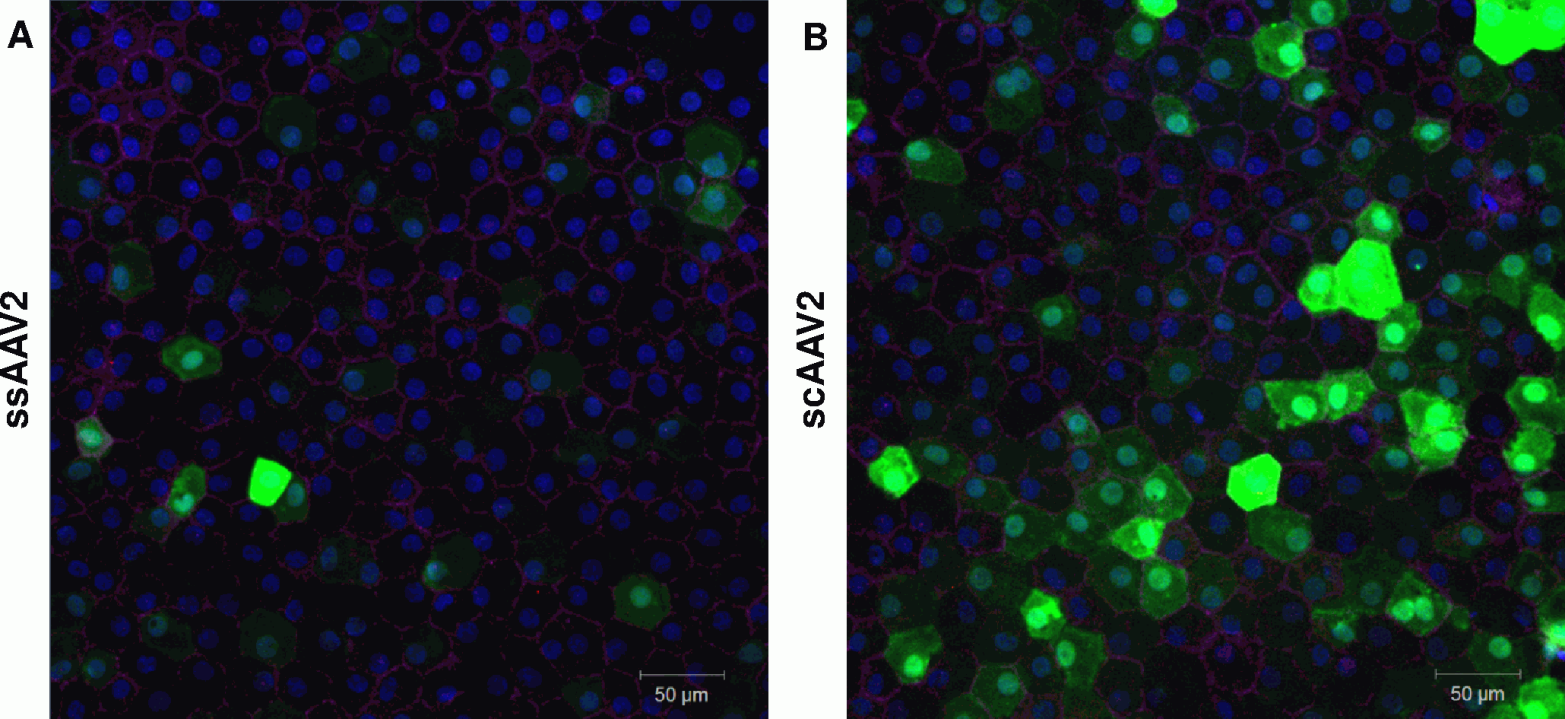
Confocal microscopy images of endothelial cells of human donor corneas six days after transduction. (A) transduced with 100,000 GOI ssAAV2; (B) transduced with 100,000 GOI scAAV2. Six random microscopic fields per corneal piece were evaluated. For each vector, one exemplary microscopic field is shown (blue = DAPI, green = GFP, pink = Alexa 647, red = TUNEL).

Six days after transduction (100,000 GOI per corneal piece) confocal microscopy showed significantly higher GFP expression in the scAAV2 group compared to the ssAAV2 group (64.7±11.3% versus 38.0±8.6%, p<0.001) (Fig 4(A) and 4(B)).

### Viability and metabolism of transduced cells

**Fig 5:**
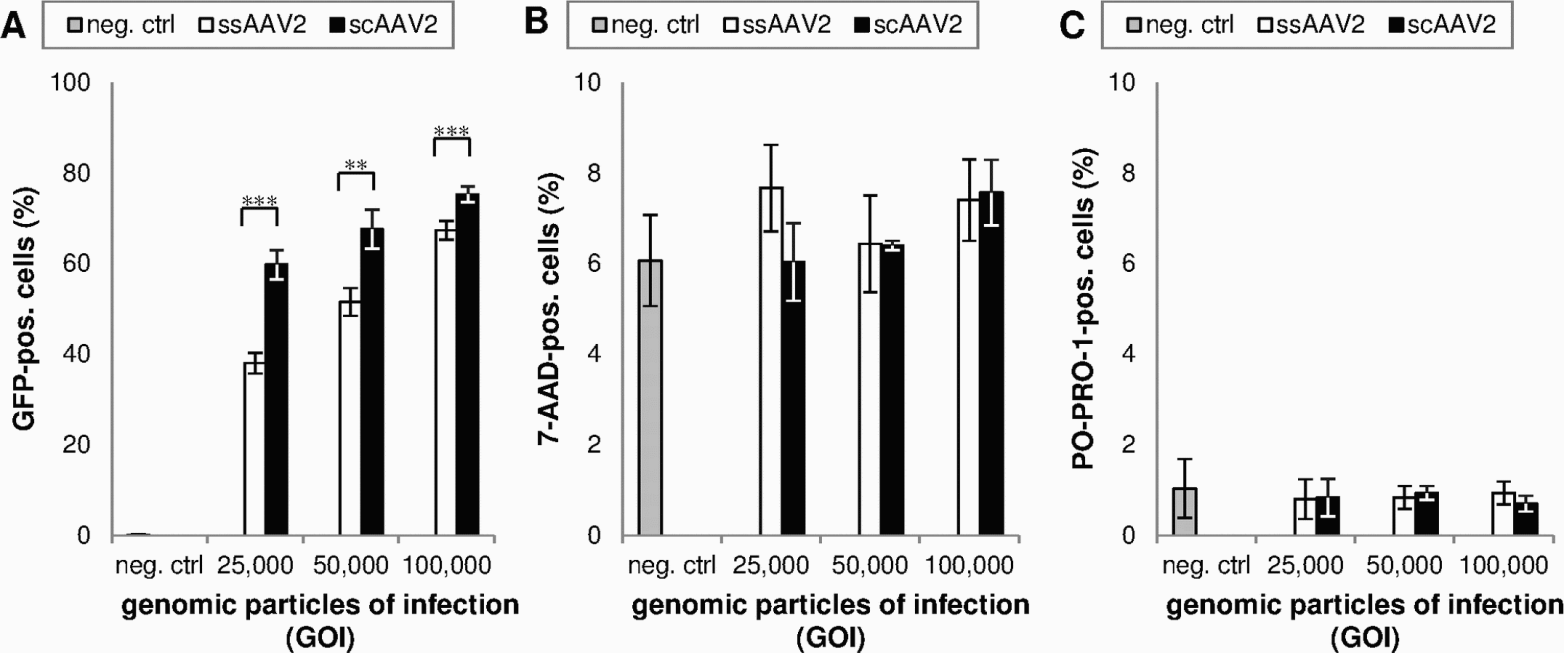
Effect of vector titer on transduction efficiency and cell viability (measured by flow cytometry two days after transduction). Means and standard deviations of three independent experiments are shown. (A) Transduced = GFP-positive cells. Transduction with scAAV2 resulted in significantly higher GFP expression rates compared to ssAAV2 (** = p<0.01, *** = p<0.001). (B) Dead = 7-AAD-positive cells. There were no significant differences between cells transduced with sc- and ssAAV2 or between transduced cells and negative controls (one-way ANOVA, p = 0.062). (C) Apoptotic = PO-PRO-1-positive cells. There were no significant differences between cells transduced with sc- and ssAAV2 or between transduced cells and negative controls (one-way ANOVA, p = 0.578).

**Fig 6:**
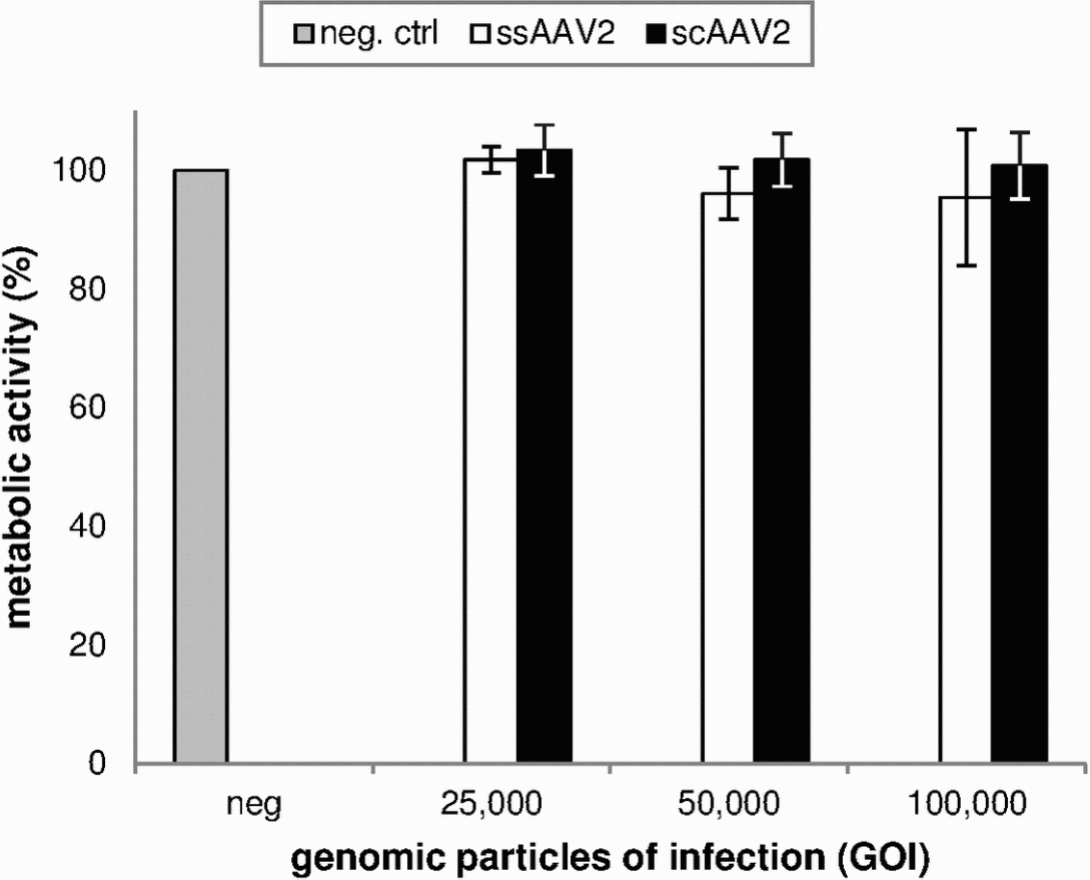
Metabolic activity of transduced cells as a sign of cell viability (analyzed by MTT-assay two days after transduction). Means and standard deviations of three independent experiments are shown. There was no significant difference between transduced cells and negative controls (one-way ANOVA; p = 0.415).

In all studied groups, there was no significant difference between cell viability of transduced and untreated cells. Neither the amount of 7-AAD positive (dead) or PO-PRO 1 positive (apoptotic) cells detected by flow cytometry (p = 0.062 and p = 0.578) (Fig 5) nor the metabolic activity quantified by MTT-test (p = 0.415) (Fig 6) significantly differed from negative control.

Confocal microscopy of human corneas transduced with ss- or sc-AAV showed regular cell morphology with intact cell membranes (visualized by ZO-1 staining). We could not detect significant DNA-strand breaks by TUNEL-assay (Fig 4).

## Discussion

For the first time, it hereby could be demonstrated that scAAV2 shows a more efficient transduction of human corneal endothelium compared to ssAAV2. Although studies have shown substantial enhancement of gene delivery by using scAAV in numerous other tissues [35–39], to date there were no data evaluating transduction of corneal endothelium with scAAV. These novel findings have significant potential to establish a gene therapeutic approach for *ex vivo* cultivated donor corneas processed in eye banks prior to transplantation.

Previous studies working on CEC and ssAAV reported transduction efficiencies ranging from 2% (rabbit and human corneas) to 96% (human corneas) [29, 30, 44]. Differences in transduction could be explained by use of different AAV serotypes as well as by different tropism in different species. In our current study, the maximum transgene expression level achieved in human corneal endothelial cells was 80.5%, compared to about 38% in one of our previous studies [29]. In earlier work of our group using a different ssAAV2 vector we observed a late onset of transgene expression and an increased GFP expression 19 days after transduction [29]. On the contrary, we could now detect remarkable levels of GFP expression as early as after two days in the same cell type. This illustrates both dependency and impact of the vector preparation (ssAAV from two different vector cores) on the gene expression outcome. Interestingly, the level of GFP expression rapidly decreased after day 5, while in the past study it continued to increase until day 23 [29]. Characteristically, CEC stop proliferation once the cells reach complete confluency because of contact inhibition. Though having used the same cell line and equal seeding conditions in both studies, it might be possible that CEC in the current experiments show a higher proliferation tendency due to a lower passage number. Therefore, with every cell division vector DNA was distributed among the two daughter cells and consequently transgene expression levels dropped over time. This, however, would not solely explain the considerable differences in onset and kinetics of GFP overexpression.

Alternatively, the use of different culture media for transduction and subsequent organ-culture might deliver an explanation: Culture medium enriched with growth factors for example leads to increased efficiency of gene delivery (96% with growth factors versus 19% without growth factors) [30]. In our study, we used a similar culture medium containing growth factors. On day 6, we observed transgene expression in 38.0±8.6% of CEC transduced with ssAAV.

To date, there are no studies evaluating the use of scAAV in corneal endothelial cells. However, in a glaucoma study aiming to transduce the trabecular meshwork of living rats and monkeys by intracameral injection of scAAV2, strong GFP expression in CEC was observed as a side effect. GFP-positivity in the corneal endothelium was present for at least 19 days after injection [25]. Unfortunately, GFP expression was not further quantified. Recently, a similar glaucoma study reported mosaic-like to almost homogenous GFP-positivity in CEC four weeks after injection of scAAV into the anterior chamber of mice and rats [26]. Transduction efficiency was described by semiquantitative evaluation of intensity and of distribution of GFP [45].

In our *ex vivo* study in human tissue, we successfully transduced 64.7±11.3% of CEC with scAAV. This represents a 1.7 fold increase relative to ssAAV. In cultured CEC, use of scAAV lead to a 4.8-fold increase in GFP-positivity compared to ssAAV. With rising vector titers, predominance in transduction efficiency of scAAV gradually diminished. These results are consistent with other studies comparing ss- and scAAV in muscle and in the retina [22, 46]. These tissues share their accessibility to local gene delivery and therefore the ability to isolate high concentrations of vector in the immediate vicinity of target cells, which facilitates high multiplicity infection [32]. This in turn increases the probability of dsDNA-formation by strand annealing or DNA synthesis [32, 47].

Transduction with scAAV not only resulted in higher, but also slightly more sustained transgene expression than transduction with ssAAV. This could be due to generally higher levels of gene expression with scAAV. Since a dsDNA genome is more stable than a ssDNA genome, which can be degraded by cellular repair mechanisms after being recognized as damaged DNA, transduction efficiency of ssAAV might be further limited compared to scAAV [48, 49].

AAV is known to be nonpathogenic and several studies have proven its safety in ocular gene therapy [50–53]. After *in vivo* transduction of CEC in rabbits no significant difference was found between central corneal thickness of transduced corneas and negative controls, suggesting that the pump function of CEC is not compromised by transduction with rAAV [31]. However, as rabbit corneal endothelium does show proliferative capacity [54–56], the suitability of findings for use in humans could be questioned. According to our histological analysis, transduction of CEC did not alter cell morphology. This is confirmed by others in histological analyses of cryosectioned, H&E stained, *ex vivo* AAV transduced human corneas [30]. The number of apoptotic cells in human corneas detected by TUNEL method did not differ significantly from negative control. Furthermore, there was no significant difference in the percentage of dead and apoptotic cells detected by flow cytometry and MTT-assay showed unchanged metabolic activity.

Moreover, in an *in vivo* glaucoma study in rats and monkeys, no humoral or cellular-mediated responses were observed in any of the transgene-expressing animals after intracameral injection of scAAV2 [25].

The use of scAAV increases transduction efficiency in comparison to their single-stranded counterparts by circumventing the rate-limiting step of second strand synthesis. This could be overcome by using higher ssAAV titers, thus increasing the probability of strand-annealing of two complementary single strands [32, 47]. However, with scAAV sufficient gene expression levels can be achieved by far lower vector titers than with ssAAV. This can be accompanied by advantageous effects: 1. increase in safety of gene delivery by reducing the risk of possible adverse effects and immune responses to the vector; 2. advances in practicability and in cost-effectiveness. A considerable drawback of scAAV seems to be its limited packaging capacity of about 2.5 kb. Therefore, the overall range of possible functional applications is limited to genes smaller than this packaging size. This aspect has to be carefully taken into account and to be weighed against the potential benefits of using scAAV [22, 32].

In conclusion, scAAV permits further optimization of AAV-mediated gene therapy of corneal endothelium, if the desired transgene does not exceed a critical size. Areas of application could include transduction of corneal allografts with anti-apoptotic genes, especially if translated to *ex vivo* treatment prior to transplantation. This would lead to the desired temporary protection of corneal endothelium during one to four weeks of cultivation in eye banks. Hence, discard rates of donor tissue being in short supply in most countries worldwide would be lowered, thus increasing transplantation figures. In addition, the rapid loss of endothelium directly following transplantation could be limited, thus lowering graft failure and re-transplantation rates. The elegant advantage of using AAV vectors is the gradual reduction of the anti-apoptotic effect within four weeks after transduction. By translating this approach into eye banking and clinic, health economic expenses for corneal transplantations could be significantly reduced.
